# Large, but Dispersal‐Limited Populations of the Marsh Fritillary *Euphydryas aurinia* Persist on Abandoned Military Training Areas Three Decades After the End of the Cold War

**DOI:** 10.1002/ece3.70459

**Published:** 2024-10-22

**Authors:** Cindy Schröer, David Singer, Johannes Kamp

**Affiliations:** ^1^ Department of Conservation Biology University of Göttingen Göttingen Germany; ^2^ Natura 2000‐Station Gotha‐/Ilmkreis Naturforschende Gesellschaft Altenburg e.V. Altenburg Germany; ^3^ Institute for Applied Animal Ecology Göttingen Germany

**Keywords:** abandonment, capture–recapture, grazing, habitat management, Jolly–Seber, Nymphalidae

## Abstract

Military training areas can host important biodiversity, due to the preservation of diverse, nutrient‐poor historical cultural landscapes and an insect‐friendly disturbance regime. In Europe, many training areas were abandoned after the end of the cold war in 1991 and the withdrawal of the Allied and Soviet forces. Many of these are now protected areas, and current management strategies vary from rewilding to active habitat management such as grazing or mowing. In a capture–release–recapture approach, marking 2418 individuals, we assessed the population size and movement patterns of the dry ecotype of the Marsh Fritillary *Euphydryas aurinia* Rottemburg 1775 on three former military training areas in Germany that varied in size and management (natural succession, mowing, and sheep‐/goat grazing). *Euphydryas aurinia* is a rare and declining butterfly species listed in Annex II of the European Union Habitats Directive. Jolly–Seber models revealed a large population of ca. 19,000 individuals on the largest study site and a smaller population at a second site, whereas recapture rates were too low to predict the population size reliably at a third site. Population densities were 190–194 butterflies ha^−1^ at the unmanaged, large site and 56–71 butterflies ha^−1^ at a smaller site grazed with sheep. Thirty‐nine percent of the recapture events occurred within the same 1‐ha‐study plot. The average minimum flight distance between the study plots was 313 m for males and 328 m for females. The maximum lifetime flight distance was 1237 m within 3 days. No dispersal was detected between study sites. Thirty years after cessation of the military use, the large former training site still held what likely is one of the largest populations of the species dry ecotype in Central Europe, including in areas where management ceased already in 1991. This suggests remarkable persistence of the species in areas without regular management, contrary to current opinion. However, regular flight distances seem not to be sufficient to connect the isolated habitat patches. It remains unknown how long the large population at the abandoned military area will persist without active habitat management. Careful, but active habitat management and restoration of habitat connectivity should thus be considered.

## Graphical abstract

We here present population sizes and movement patterns of the endangered Marsh Fritillary based on an large‐scale and very intensive capture–release–recapture study conducted on three former Central European military training areas. Our results suggest that against common belief, very large populations of the Marsh Fritillary can survive decades after land abandonment in former military training areas. However, our results point also to a very low mobility, which suggests that conservation measures might not only need to involve habitat management but also increase habitat connectivity.

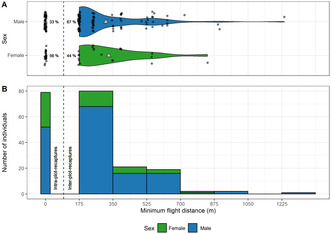

## 1 Introduction

Insects are declining, with some studies suggesting dramatic losses in different parts of the world (Hallmann et al. 2017; Seibold et al. 2019) and a net negative trend for terrestrial insect abundance and biomass worldwide (van Klink et al. 2020). Other analyses have painted a more nuanced picture, with declines, increases, and stable trends across regions, taxa, and traits (Crossley et al. 2020; Neff et al. 2022). Overly alarming messages have also been put into perspective by authors suggesting a need to account for several methodological problems (Daskalova, Phillimore, and Myers‐Smith 2021; Mupepele et al. 2019). Nevertheless, there is evidence for worrying declines of many taxa, with potential consequences for entire ecosystem services and humanity (Cardoso et al. 2020).

Many butterfly species decline, especially in Europe and Asia (van Swaay et al. 2012). Here, important drivers of butterfly declines are agricultural intensification (Habel et al. 2019), the abandonment of cultural landscapes following socioeconomic change (Daskalova and Kamp 2023; Colom, Traveset, and Stefanescu 2021; Mora, Wilby, and Menéndez 2022; Trappe et al. 2017), the loss of light forests due to changes in forest management and policies (van Swaay et al. 2012), and climate change (Hill et al. 2021). The European Grassland Butterfly Indicator that builds on data from 16 European countries suggests a 39% decline of grassland butterflies since 1990 (Warren et al. 2021). Abundance losses started long ago (Laussmann, Dahl, and Radtke 2021), with declines in the area of occupancy of up to 80% since the late 19th century (Warren et al. 2021) and distinct periods of loss linked to changing societies and land use patterns (Habel et al. 2022).

Butterflies are often in the focus of conservationists, as they are relatively easy to identify and survey, perceived as beautiful by the general public and good ecological indicators. Despite the high vulnerability of butterflies to different global change drivers and their popularity, there is a dearth of information on population densities, basic ecological traits, and variables such as dispersal, habitat selection, and demographic information for many species. Ecological knowledge is increasingly synthesized across larger scales allowing to derive conservation policies on various spatial levels from regional to global (Warren et al. 2021). In contrast, labor‐intensive and financially demanding field studies shedding light on the natural history of species seem to be increasingly less‐valued. This is unfortunate, as exactly this natural history information is needed to advise conservation authorities and habitat managers on which measures are needed to reverse the fortune of declining species, especially for species of supranational conservation interest such as those listed in the annexes of the European Union's Habitats Directive (van Swaay et al. 2012).

In an era of pan‐European butterfly declines, only few areas exist that provide beneficial conditions for survival and persistence. Protected areas can be effective in preserving butterfly diversity (Cizek et al. 2013). Many butterfly species require ecological disturbance (canopy opening, opening of dense grass swards by mechanical disturbance, grazing, or fire) to allow their foodplants to thrive, and for suitable microclimate, large and important butterfly populations also thrive in limestone quarries and open‐cast mines (Beneš, Kepka, and Konvička 2003; Münsch and Fartmann 2022), and on active military training areas (Smith, Turner, and Rusch 2002; Konvička et al. 2021). In these secondary habitats, in addition to frequent disturbance (Warren and Büttner 2008), historical cultural landscape elements have also been preserved here that were abandoned, intensified and transformed elsewhere, such as heathlands, sand dunes, and unfertilized grasslands. Often, the conservation value of military training areas is therefore similar to or higher than those of established protected areas (Cizek et al. 2013).

In Europe, large military training areas were established prior to and during the two world wars and continuously used during the cold war. After the end of the cold war in 1989, both small and very large military training areas of both the Western Allies and the Soviet and Eastern Block forces were either abandoned or repurposed in the 1990s, e.g., for solar and wind parks or recreational use (Franz and Szendiuch 2014). Acknowledging their rich biodiversity and their importance for conservation, many sites in Europe were also registered as Natura 2000 sites (Ellwanger and Reiter 2019). Some of them are managed to maintain the open character of grassland and heathland (Konvička et al. 2021), but at many sites, management ceased altogether, intentionally or unintentionally, initiating vegetation succession and reforestation. This has often resulted in biodiversity loss (Zografou et al. 2017), but the full scale of this abandonment and its implications for biodiversity are insufficiently documented.

While rewilding of abandoned military training areas has led to increasing population sizes of large carnivores such as wolves (Reinhardt et al. 2019) and positive responses of grasshoppers (Reif et al. 2023), vascular plants (Reif et al. 2023), and some bird species (Culmsee et al. 2021; Reif et al. 2011), a cessation of military training usually led to only initial increases, but long‐term losses of many arthropod species, including butterflies (Reif et al. 2023; Zografou et al. 2017). Among these are some listed in Annex II of the European Union's Habitats Directive that aims to protect vulnerable and threatened habitats and species (Directive Habitats 1992).

The Marsh Fritillary *Euphydryas aurinia* Rottemburg 1775 has been declining rapidly across Central and Northern Europe, with associated large‐range contractions. It is hence listed in Annex II of the European Union's Habitats Directive, despite being still widespread in other parts of Europe (European Red List status “least concern,” van Swaay et al. 2012). Declines were first noticed in the UK, with a loss of 62% of the known populations between 1983 and 1990 (Warren 1994). The species was formerly widespread in Denmark but is now found only at a single site (Brunbjerg et al. 2017). In Germany, the species' range contracted by 75% between 1950 and 2002 (Anthes et al. 2003). The reasons for these declines are habitat loss due to land abandonment and unsuitable management (van Swaay et al. 2012) but also increasing habitat fragmentation and genetic isolation (Pertoldi et al. 2021).

The Marsh Fritillary inhabits either dry, calcareous grasslands or damp, acidophilous grasslands. The larvae feed on ca. 20 different plant species (Anthes and Nunner 2006) but by far prefer *Scabiosa columbaria* in the dry ecotype. The larvae of the wet ecotype feed mostly on *Succisa pratensis*. Populations can switch between wet and dry habitats: In Thuringia, our study region, the species inhabited exclusively wet habitats until ca. 1980 (Thust et al. 2006) but then started colonizing large areas of dry habitat in less than a decade in explosive expansion (Thust et al. 2006; Rommel and Schäfer 1999), before new declines set after the year 2000 (Anthes et al. 2003). Warren (1994) suspected that the dry habitats have a generally lower habitat suitability, and appropriate management is more difficult to implement.

We surveyed populations of the dry ecotype of the Marsh Fritillary on three former military training areas used by the Soviet Red Army during the cold war in Thuringia, central Germany. The Soviet forces withdrew in 1991 and 1992. The sites vary in current management and management history, which ranges from abandonment followed by vegetation succession to livestock grazing to maintain open swards.

We aimed:

1. To assess current population sizes and population densities in the remaining large Central European population, across differently managed former military training areas.
2. To assess movement distances and movement patterns to inform the scale of conservation planning needed for the species.
3. To gather evidence of long‐distance movements potentially connecting the studied populations.

## 2 Methods

### 2.1 Study Sites

We surveyed butterflies across three study sites in Thuringia, central Germany that were known to host populations of *E. aurinia* (Figure S1). All surveyed sites are parts of former military training areas from which the Soviet Red Army withdrew in 1991/1992. Most of their area is now registered as Special Areas of Conservation (SAC) under the EU Habitats Directive. The climate is continental, with a mean annual precipitation of 600–800 mm and a mean annual temperature of 7°C–8°C (Schleip 2017; Thüringer Landesanstalt für Umwelt und Geologie 2017).

The study site National Park “Hainich” comprises ca. 7500 ha. A first military training area (“Kindel”) was established in 1935 and extended in 1961–1964. Military use was abandoned in 1992, and the area is a SAC since 1997. We focused on 2000 ha successional grassland in the south of the park (Figure S2). The national park is divided into a strict conservation zone, where no management is undertaken and former open grassland transitions into shrub and forest. In a buffer zone, grasslands are managed by mowing or rotational grazing. The study site “Krahnberg—Kriegberg” (henceforth Kriegberg) had been used as military training area since the late 19th century, was last used by the Soviet Red Army in 1992, and was registered as SAC in 2004. We focused on a 35‐ha calcareous, seminatural dry grassland area inside the SAC (Figure S3). The area is grazed with sheep and goats. The study site “Truppenübungsplatz Ohrdruf‐Jonastal” (henceforth Jonastal) is a large training area that has been used by the military since 1908 and is still in use by the German armed forces. It has a total area of ca. 6000 ha and was registered as a SAC in 2004. We focused here on a smaller area of 26 ha that was taken out of military use in the early 1990s (Figure S3). The area is characterized by seminatural dry grasslands on calcareous soils and managed by grazing and mowing. Information on designation time, management, and characteristics of all SACs can be downloaded from https://natura2000.thueringen.de/download‐bereich.

### 2.2 Butterfly Surveys

As one major aim was to estimate site‐specific population sizes at all three study areas, we first identified the currently known distribution of *E. aurinia* based on previous survey data (Czarnowsky 2016) and further potential occurrences based on known areas with the larval food plants. Those parts of the study sites that were identified as potentially suitable habitat or known to host the study species were overlaid with a 175 m × 175 m point grid in QGIS 3.4.10. Each point served as a centroid of a square with 100 m site length, resulting in sampling plots of 1 ha. Such grid‐based methods allow a standardization of sampling effort and have been shown to lead to higher detection probability in *E. aurinia* (Norman et al. 2024). As a result, these plots were separated from each other by a 75 m strip that was not surveyed. Plots that contained more than 50% habitat considered unsuitable for the species (e.g., forest) or were covered less than 50% by the protected area were excluded. This resulted in 106 surveyed plots at the study site Hainich, 15 at Kriegberg and 10 at Jonastal (Figures S1–S3).

All plots were visited 12 times between 16 May 2020 and 09 June 2020. Two site visits were conducted on two consecutive days; afterward, we paused for 1–4 days before visiting a site again. On each sampling day, the complete set of plots was visited by seven out of eleven experienced observers. Each plot was intensively searched for imagines of *E. aurinia* for 10 min, equaling a total search effort of 120 min per ha. For each individual captured, 2 min was added for handling time (capture, sex determination, marking, and data entry), with a possible maximum of two time extensions. The GPS location of each capture event was recorded with the Locus GIS 1.7.0 app on Android smartphones. Recaptures at the same day in the same plot were not recorded. All imagines were marked individually with a dark permanent marker on the underside of the wing and sexes distinguished were possible. Surveys were only conducted at an ambient air temperature between 15°C and 22°C and wind speed below 20 km/h (DWD 2024) (Table S1).

### 2.3 Data Analysis

Data were analyzed using R 4.4.0 (R Core Team 2024) accessed through R‐Studio (Posit Team 2023). For the analysis of movement distances, the data of all three study sites were combined (*n* = 195 individuals with 206 recapture events). Flight distances were calculated as the shortest line between the centroids of the sampling plots of the consecutive capture events of all individuals, with the UTM zone 32N (EPSG: 25832) as a coordinate reference. We distinguished between recaptures within and between plots. The minimum possible flight distance of interplot recaptures was ≥ 175 m due to the 75 m buffer zone between sampling plots (see above). Each recapture event was considered an independent data point for the analysis of minimum flight distances. To infer movement patterns, we counted the inter‐ and intraplot recaptures for all combinations of plots and visualized the connections within and between plots on a map.

We estimated local population sizes (*N*) with Jolly–Seber models in MARK version 6.2 using the POPAN parameterization (White and Burnham 1999) and accessed through the package RMark (Laake 2013). Survival probability (phi), recapture probability (*p*), and the probability of individuals entering the population through birth or immigration (pent) were estimated. All model parameters were included as time‐dependent (both linear ~ Time and quadratically ~ Time + I(Time^2)) and time‐independent (~1); recapture probability was also included as a function of weather (~temperature, ~windspeed, ~sunshine duration) as well as the combination of these weather parameters. We ran the models for all individuals as well as for males and females separately. Weather data at the survey dates were taken from the DWD weather station Eisenach (DWD 2024), which is the closest station to all three sites. Mean temperature, wind speed, and total sunshine duration were calculated for the daily sampling periods (9 a.m. to 5 p.m.) (Table S1). The final population size was predicted from the model with the lowest AIC_c_ value. Where *p*, phi, and/or pent of the best model were assumed time‐ or weather‐dependent, the mean of the calculated values and the associated standard error and 95% parametric confidence interval were calculated. A goodness‐of‐fit test for the models was conducted for the sites Hainich and Kriegberg, using the function overall_CJS in R package R2ucare (Gimenez et al. 2018). The residence time was defined as the timespan between the first and the last capture event per individual (*n* = 195 recaptured individuals) and calculated using the lubridate R package (Grolemund and Wickham 2011). We note that residence time as defined here is governed by both apparent survival and recapture probability. Population densities were calculated by dividing the estimated number of individuals by the total area of plots that had at least one capture. Hence, densities are based on the estimated minimum distribution of the species in the areas.

## 3 Results

At the Hainich site, 2207 individuals were caught and marked, of which 172 were recaptured once and eight were recaptured twice (recapture rate of 8.2%). At Kriegberg, 154 individuals were marked, and 20 recaptured at one to three sampling events (recapture rate 13.0%), whereas at the Jonastal site, 57 individuals were marked and three of these recaptured once (recapture rate, 5.3%) (Table S2). Recaptures at two consecutive sampling events were made only for nine individuals, at three only for one individual. At all study sites, fewer females than males were captured (Table S2).

**FIGURE 1:**
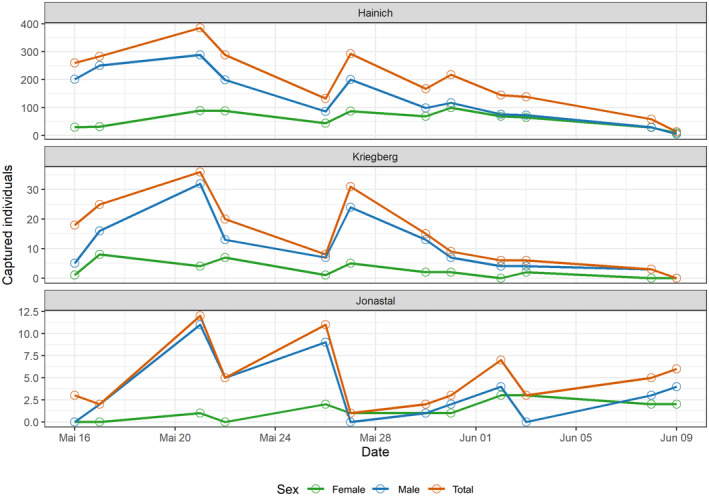
Total captured Marsh Fritillaries per sampling event over the study season 2020 at the three study sites, separately for both sexes and combined.

The phenology was sex‐dependent, with males generally hatching earlier and females appearing later (Figure 1).

**FIGURE 2:**
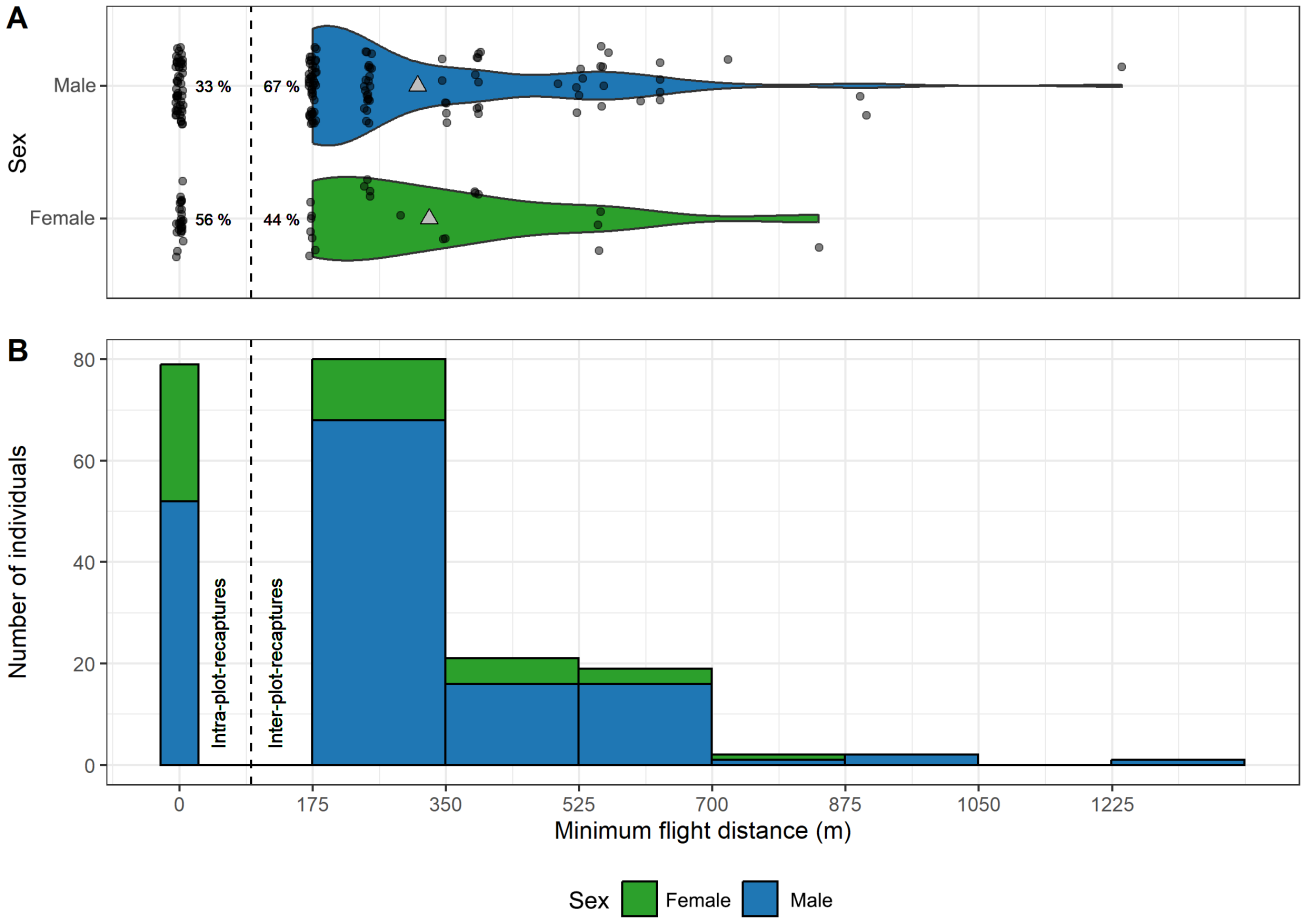
Distribution of minimum flight distances of the Marsh Fritillary within (“intraplot recaptures”) and between (“interplot recaptures”), shown as violin plots for both sexes (panel A) and histogram (panel B). Triangles in panel A indicate the mean of the minimum flight distance of interplot recaptures.

There were 206 recapture events in total at all three study sites. Eighty (39%) of the recaptures occurred within the same plot, while in 126 (61%) cases, individuals were recaptured at least in a neighboring or even more distant plots. The proportion of intra‐ and interplot recaptures differed significantly between sexes (Pearson's Chi‐squared test, *χ* ^2^ = 7.187, df = 1, *p* = 0.007). Fifty‐six percent of the recaptures of females occurred within the same plot, while for males, only 33% of the recaptures were recorded within the same plot (Figure 2). For the interplot recaptures, minimum flight distances were not significantly different between females and males (Wilcoxon test, *W* = 1096.5, *p* = 0.979). The average minimum flight distance of these interplot recaptures was 318 (±192 SD) m. The average minimum flight distance of the inter plot recaptures was 313 m (±193 SD) for males and 328 m (±176 SD) for females (Figure 2). The maximum flight distance was 1237 m and was covered by a male in three days at Hainich national park.

**FIGURE 3:**
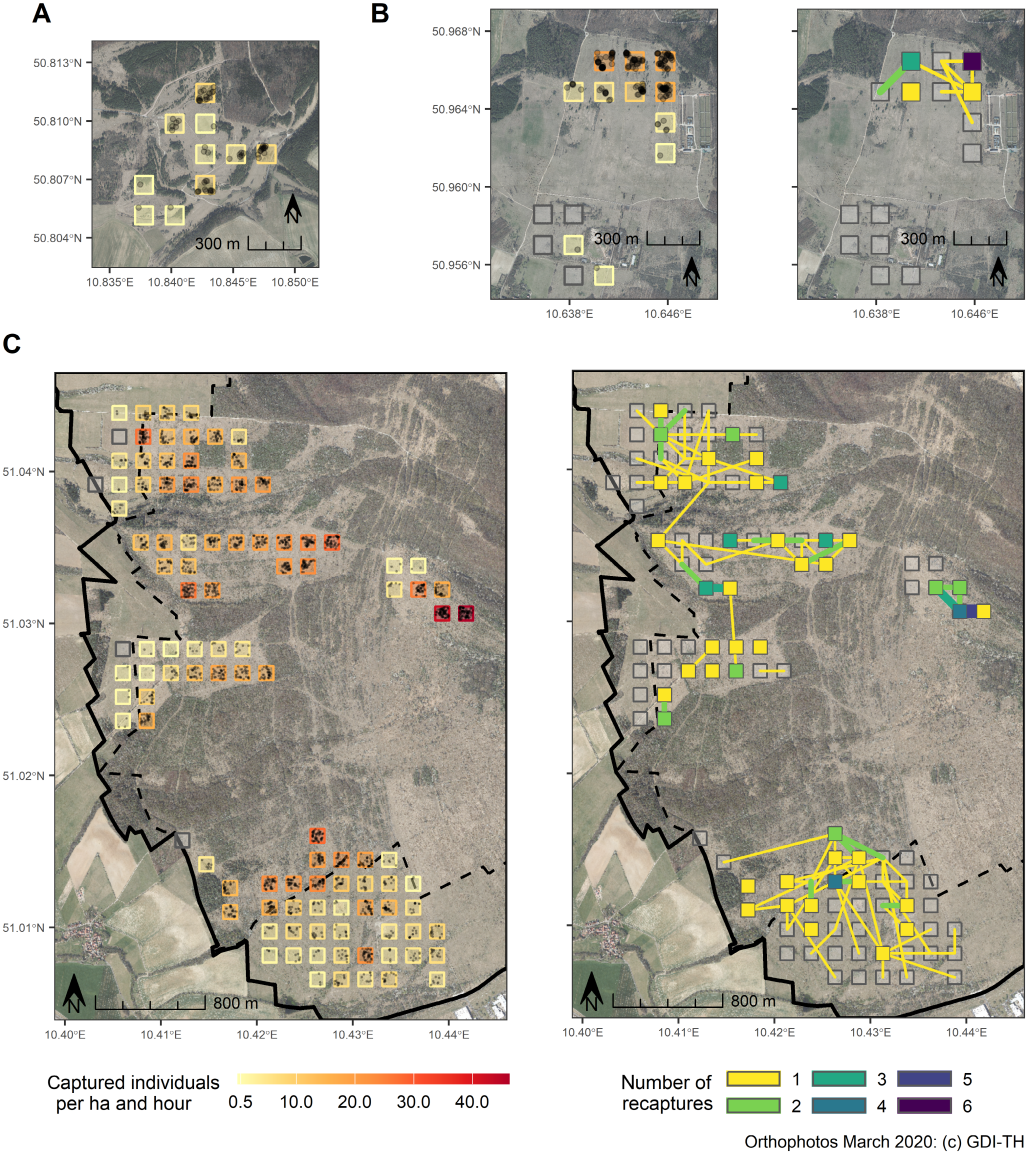
Observed density of captured individuals ha^−1^ and number of recaptures between (colored lines) and within (colored plot area) plots at the study sites Jonasberg (panel A), Kriegberg (panel B), and Hainich (panel C). The solid line at the Hainich site shows the border of the national park, and the dashed line designates the core zone of the national park without habitat management. Black dots are individual capture events.

The spatial distribution of the recaptures indicated variation in connectivity of the study plots. No exchange between the southern and northern parts of the Hainich site was recorded while there was no recapture at all in the southern part of the Kriegberg site (Figure 3). No exchange of individuals was detected between study sites.

The mean residence time of recaptured individuals was 5.8 days (±4.6 SD) with a maximum of 18 days. The average residence time of females (3.8 days ± 1.32 SD) was significantly shorter than that of males (6.3 days ± 4.7 SD) (Wilcoxon test, *W* = 2346, *p* = 0.002).

Models for the Jonastal site showed a lack of fit and evidence for spurious parameter estimates, likely due to low sample size. We therefore do not present population size estimates for this site. The same applies for the sex‐specific models at the Kriegberg site.

**FIGURE 4:**
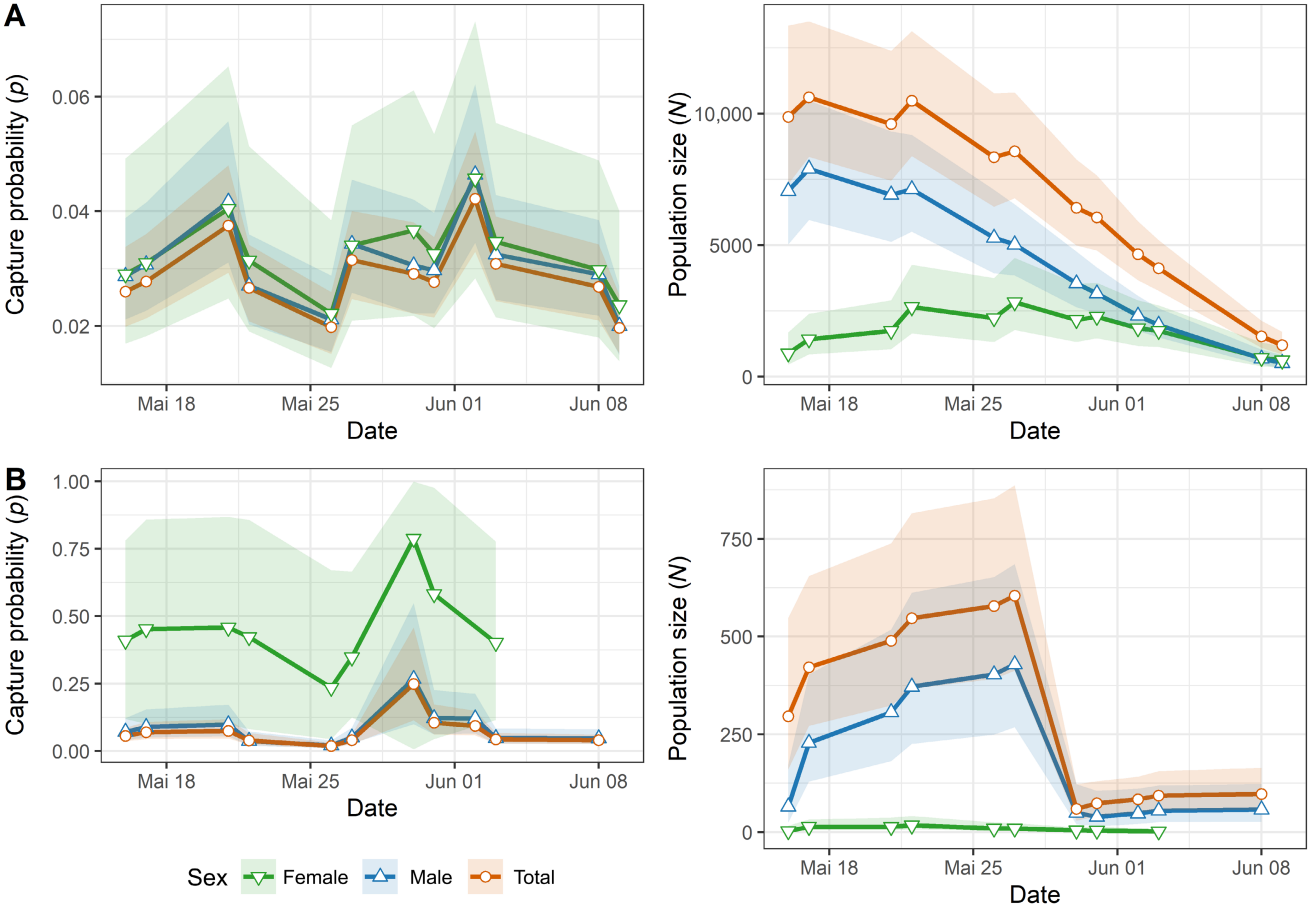
Daily estimates and their confidence limits from the best population model (cf. Table 1) of *Euphydryas aurinia* at the Hainich nationalpark site (A) and Kriegberg (B) in 2020. Estimates of capture probability *p* and population size *N* are shown for sexes separately and for the total population (including a small number of individuals with unknown sex).

For the large site (Hainich), the best model assumed survival probability phi to be time‐dependent, recapture probability *p* to be affected by temperature, wind speed and sunshine duration and the probability of entering the population through immigration/birth pent to be time‐dependent (quadratically) (Table 1). The population size estimate from this model was 19,579 (CI: 16,861–22,801) individuals. There was one more model that had ΔAIC of less than 2 compared to the best model (Table 1). This model also assumed an effect of temperature, wind speed, and sunshine duration on recapture probability. Beta estimates provide evidence of a positive effect of all weather estimates on recapture probability. All further models had ΔAIC of > 3.5 compared to the best model (Table 1). Sex‐specific models revealed that females reached their population peak at Hainich during the sampling period, while for males the population was probably already at maximum during the first sampling days. Recapture probability *p* varied throughout the sampling period but not between sexes (Figure 4).

**TABLE 1:** Population model summaries for the study sites Hainich and Kriegberg: Population size *N* [95% confidence intervals], number of model parameters *npar*, AIC_c_ values, ΔAIC compared to the model with lowest AIC_c_, Akaike weights, and density of individuals ha^−1^ [95% confidence intervals].

	*N*	*npar*	AIC_c_	ΔAIC	Weight	Individuals ha^−1^
*Hainich model*						
Phi(~Time)*p*(~temp + wind + sundur)pent(~Time + *I*(Time^2))*N*(~1)	19,579 [16,861–22,801]	10	1871.07	0	0.556	192 [165–224]
Phi(~Time + *I*(Time^2))*p*(~temp + wind + sundur)pent(~Time + *I*(Time^2))*N*(~1)	19,341 [16,584–22,627]	11	1872.72	1.649	0.244	190 [163–222]
Phi(~Time)*p*(~temp + wind + sundur)pent(~Time)*N*(~1)	19,446 [16,732–22,666]	9	1874.65	3.580	0.093	191 [164–222]
Phi(~Time + *I*(Time^2))*p*(~temp + wind + sundur)pent(~Time)*N*(~1)	19,768 [16,958–23,113]	10	1875.69	4.620	0.055	194 [166–227]
*Kriegberg model*						
Phi(~Time + *I*(Time^2))*p*(~sundur + wind)pent(~Time)*N*(~1)	661 [468–971]	9	271.73	0	0.384	60 [43–88]
Phi(~Time + *I*(Time^2))*p*(~temp + wind + sundur)pent(~Time)*N*(~1)	643 [454–951]	10	273.21	1.480	0.183	58 [41–86]
Phi(~Time + *I*(Time^2))*p*(~Time)pent(~Time + *I*(Time^2))*N*(~1)	712 [477–1117]	9	274.47	2.740	0.098	65 [43–102]
Phi(~Time + *I*(Time^2))*p*(~sundur + wind)pent(~1)*N*(~1)	634 [450–934]	8	274.64	2.902	0.090	58 [41–85]
Phi(~Time + *I*(Time^2))*p*(~temp + wind + sundur)pent(~Time + *I*(Time^2))*N*(~1)	777 [522–1208]	11	275.37	3.633	0.062	71 [47–110]
Phi(~Time + *I*(Time^2))*p*(~temp + wind + sundur)pent(~1)*N*(~1)	616 [616–616]	9	275.49	3.759	0.059	56 [56–56]
Phi(~Time + *I*(Time^2))*p*(~sundur + wind)pent(~Time + *I*(Time^2))*N*(~1)	726 [495–1113]	10	275.81	4.074	0.050	66 [45–101]
Phi(~Time + *I*(Time^2))*p*(~temp + sundur)pent(~Time + *I*(Time^2))*N*(~1)	670 [471–992]	10	278.08	6.351	0.016	61 [43–90]

*Note:* Only the models up to a cumulative weight of 0.95 are shown.

For the smaller site (Kriegberg), the best model assumed phi to be time‐dependent (quadratically) and *p* to be affected by sunshine duration and wind speed, while pent is time‐dependent (Table 1). The population size estimate *N* from this model was 661 (CI: 468–971) individuals. There was one model within two points of ΔAIC, showing an impact of weather on recapture probability (Table 1). Beta estimates provide evidence of a positive effect of all weather estimates on recapture probability.

The estimated population sizes translated into population densities of 201 (CI: 173–233) individuals ha^−1^ at Hainich and 70 (CI: 48–104) individuals ha^−1^ at Kriegberg. Chi‐square goodness‐of‐fit tests revealed no evidence for a lack‐of‐fit of the used models (Kriegberg: *χ* ^2^ = 4.136, df = 17, *p* = 0.999, Hainich: *χ* ^2^ = 27.902, df = 37, *p* = 0.86).

## 4 Discussion

We here show with an extensive capture–recapture study that the Europe‐wide declining Marsh Fritillary *E. aurinia* persists with very large populations on former military training sites in Central Europe 30 years after their abandonment by the Soviet Red Army. However, the mobility of these populations was low with 39% of recaptures recorded at the same plot, a mean minimal flight distance between adjacent plots of 318 m, a maximum lifetime flight distance of 1237 m within 3 days, and no long‐distance movements between study sites detected despite the marking of over 2400 individuals.

The population densities estimated in our study (56–71 individuals ha^−1^ at the Kriegberg site and 190–194 individuals ha^−1^ at Hainich) are within the range of vital populations found in other parts of Europe, e.g., 32 ind. ha^−1^ in the Westerwald area/Germany (Fischer 1997), 34 ind. ha^−1^ in the UK (Warren 1994), 80–120 ind. ha^−1^ in Czech Republic (Zimmermann et al. 2011), and 232 ind. ha^−1^ in Slovenia (Jugovic et al. 2018), but below densities found in prime habitat in Romania (570 ind. ha^−1^, Junker, Rákosy, and Schmitt 2023). However, regarding absolute population size, the population at Hainich National Park with ca. 19,000 individuals is likely one of the largest populations of *E. aurinia* in Central Europe as it provides very large expanses of suitable habitat.

The Hainich site has been abandoned by the military and is since not managed over its largest parts, deliberately allowing vegetation succession, shrub encroachment, and reforestation under a policy of natural development and process‐oriented conservation. Due to lack of baseline data from before 1991, we cannot rule out that the population has declined since the military training area was abandoned. However, given the population densities at the large Hainich site, it is unlikely that declines were severe, and indeed likely that the population has increased since the abandonment of the site, as the dry habitat ecotype of the species started to appear in Thuringia only after 1980. The persistence is remarkable, given that the cessation of management, subsequent litter accumulation, and a changing microclimate are often held responsible for butterfly declines, including in Marsh Fritillary (Scherer and Fartmann 2022). Soil compaction by tanks has slowed down the succession process sustainably when the training area was used actively (cf. Guretzky, Anderson, and Fehmi 2006). Furthermore, large mammals like wild boars and deer are very abundant at the Hainich national park, actively decelerating reforestation of the unmanaged areas. Their foraging behavior disturbs the vegetation, which can promote larval foodplants of rare butterfly species (Labadessa and Ancillotto 2023), including in our study population at the Hainich site (Scherer et al. 2024).

**FIGURE 5:**
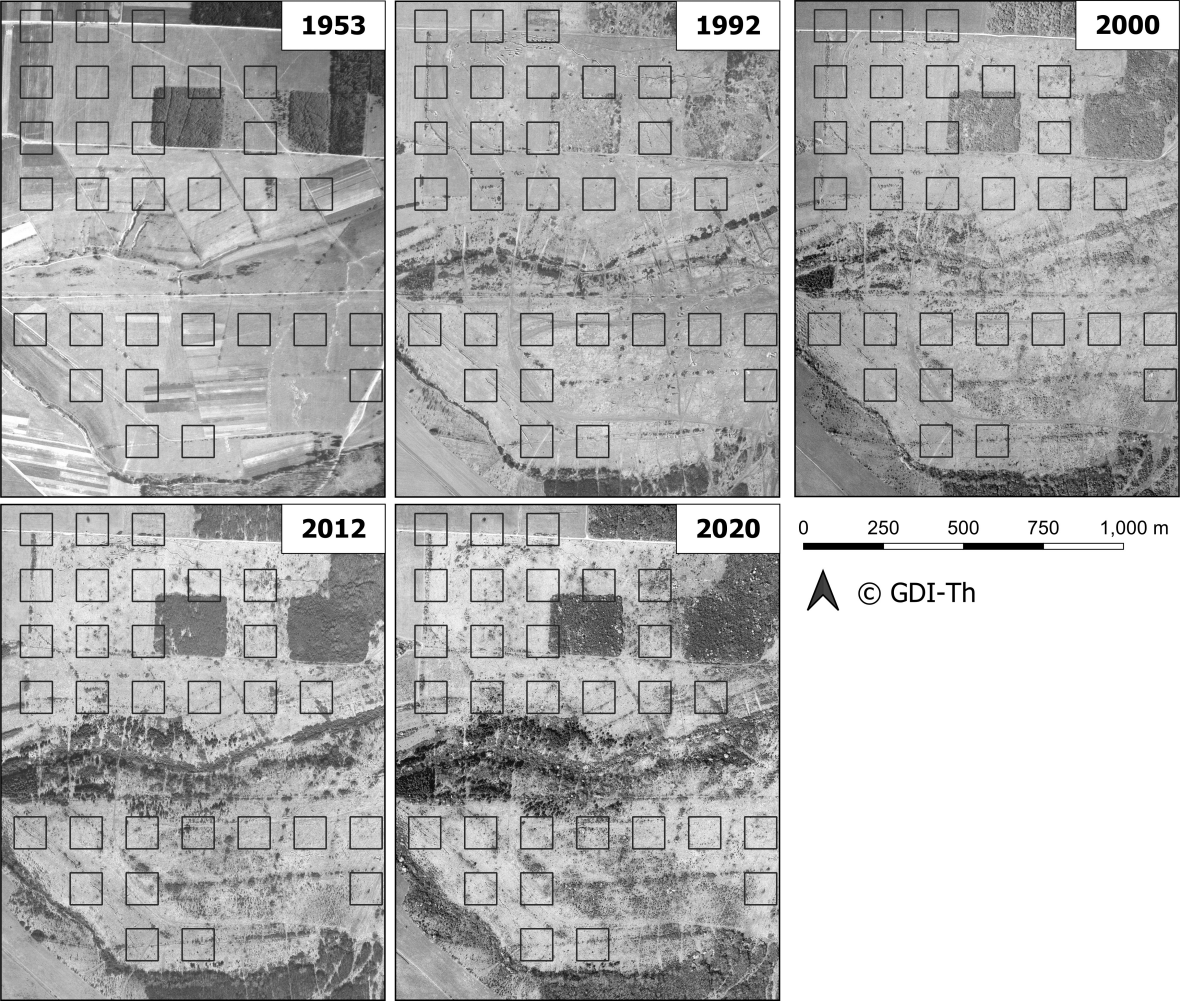
Time series of historical aerial photographs/orthophotos of a part of the study area at Hainich National Park, downloaded from the open geodata repository of Thuringia (GDI‐Th). An increasing shrub vegetation succession can be observed since the abandonment of the military training area in 1992.

Historical aerial photos indicate that the area covered by shrubs and trees has been increasing since the abandonment of the military training area at Hainich in 1992 (Figure 5). It is unclear how long the large population at the abandoned military areas will persist without active habitat management, but a quick disappearance has already been predicted 20 years ago (Thust et al. 2006). Populations of *E. aurinia* may well have already run up an extinction debt, as shown earlier for the species in the UK (Bulman et al. 2007).

Abundance was much lower at the managed sites, which might perhaps indicate suboptimal management. In Kriegberg and Jonastal, sheep and goats graze, while at the managed parts of the Hainich site, management varied between fenced sheep and goats and mowing. Sheep prefer herbs and disdain old grass (Grant et al. 1985; Thoss et al. 2005), which might lead to a selective reduction of nectar and larval food plants and loss of larval stages.

The low mobility we report here is also characteristic for other areas: Mean lifetime movements were 265 m in males and 154 m in females in Czech Republic (Zimmermann et al. 2011) and 99.4 vs. 67.3 m in high‐quality habitats in Romania (Junker, Rákosy, and Schmitt 2023). Maximum distances of single movements were 349 m (males) and 283 m (females) in Portugal (Junker and Schmitt 2010), but longer movements of more than 4 km between isolated populations were detected in a national‐scale study in Czech Republic (Zimmermann et al. 2011). As our study sites are located more than 15 km apart from each other and mainly surrounded by intensively used agricultural areas, gene flow seems currently unlikely between the populations. However, also on the local scale at Hainich national park, our data indicates an isolation of parts of the local populations, likely due to shrub encroachment acting as flight barriers after abandonment of the military training area (Figure 5). *E. aurinia* is known to be sensitive to isolation of habitat patches (Scherer and Fartmann 2022). This suggests that corridors might be needed, cut within large expanses of overgrown shrubby and reforested areas to facilitate more exchange within local populations. Likewise, corridors between the three studied sites and other potentially suitable sites where the species vanished could be established to stimulate dispersal. These should provide permeable habitat, but not at too high habitat quality, as individuals might not move if the habitat quality is too high (Haddad and Tewksbury 2005). Long‐distance flights are dangerous and energy‐intensive, thus undertaken seldomly by few individuals. Scarcity of resources and small, isolated habitat patches can promote dispersal (Baguette 2003; Baguette and Schtickzelle 2006; Schtickzelle, Mennechez, and Baguette 2006), which might be a reason for the rather short flight distances detected in this study.

In our study, recapture rates were low (5%–13%), compared to rates of 31%–54% from Slovenia (Jugovic et al. 2018), China (Wang et al. 2004), Germany (Ulrich 2004), and Romania (Junker, Rákosy, and Schmitt 2023). For the Hainich site, it is likely that the enormous population size led to few recaptures; thus, the recapture rate might increase with even higher sampling effort. For the other two study sites, we can only assume that larval and adult habitat might differ; thus, individuals left the study plots for mating and egg laying.

Implications for conservation: Given the low mobility and ongoing reforestation of the sites, active habitat management might be needed to maintain the populations, which are likely of Europe‐wide importance. Management comprises, mostly for logistical reasons, sheep grazing in a part of the site (e.g., Figure S2). However, sheep are managed by rotational grazing and therefore fenced at high density for longer periods, often depleting larval and adult resources of Marsh Fritillary at a critical time. It is currently unknown how this affects the population, but there are suggestions that year‐round grazing with low stocking densities of cattle or horses as in other former military training areas in Central Europe (Köhler et al. 2023) might also benefit butterflies in our study areas. Trials at the Hainich national park have included fencing off a bigger, 112 ha area instead of smaller patches in 2021, allowing sheep and goats to roam over a larger area in lower densities, with associated monitoring of the outcomes.

The sensitivity of *E. aurinia* to mowing varies between regions (Schwarz et al. 2023), and many populations have been doing well on mown sites over long periods (Anthes and Nunner 2006). At the managed parts of Hainich national park, rotational mowing is implemented in mid‐June, early enough to not affect the hibernation webs. In 2022, the mowing technique was changed to cutter‐bar with a minimum cutting height of 10 cm, known to be more insect‐friendly than other techniques (Gorthner 2021; van de Poel and Zehm 2015; Humbert, Ghazoul, and Walter 2009).

The survival of the here‐studied, exceptionally large remaining population of an endangered species of the EU Habitats Directive within the Hainich national park will likely depend on a prudent concept of various management measures to achieve balance between allowing natural succession and maintaining vegetation disturbance. Therefore, a relocation of the strict protection zone at Hainich national park seems mandatory to preserve the favorable conservation status of this population of *E. aurinia*.

## Author Contributions

**Cindy Schröer:** conceptualization (equal), data curation (equal), formal analysis (equal), funding acquisition (lead), investigation (lead), methodology (equal), project administration (lead), writing – original draft (lead), writing – review and editing (equal). **David Singer:** conceptualization (equal), data curation (equal), formal analysis (equal), methodology (equal), software (lead), visualization (lead), writing – review and editing (equal). **Johannes Kamp:** conceptualization (supporting), supervision (lead), writing – original draft (equal), writing – review and editing (equal).

## Conflicts of Interest

The authors declare no conflicts of interest.

## Supporting information


**Figure S1.** Overview of the location of the three study areas in Thuringia.
**Figure S2.** Sampling design, observations of *Euphydryas aurinia* and management of the National Park “Hainich”.
**Figure S3.** Sampling design, observations of *Euphydryas aurinia* and management of the sites Kriegberg und Jonastal.
**Table S1.** Weather parameters at the survey dates, measured at the DWD weather station Eisenach (DWD 2024).
**Table S2.** Total number of marked individuals of *Euphydryas aurinia*, percentage of sexes and number of recaptured individuals per study site.

## Data Availability

The data and R‐code to reproduce the analysis are openly available at https://github.com/d‐singer/aurinia_thuringia.git.
