# Supplementary material for: Large, but Dispersal‐Limited Populations of the Marsh Fritillary Euphydryas aurinia Persist on Abandoned Military Training Areas Three Decades After the End of the Cold War
Source: Ecol Evol. 2024 Oct 22;14(10):e70459. doi: 10.1002/ece3.70459 (PMC11494246; doi:10.1002/ece3.70459)
Supplement: Supplementary file 1 — Figure S1. Overview of the location of the three study areas in Thuringia. Figure S2. Sampling design, observations of Euphydryas aurinia and management of the National Park “Hainich”. Figure S3. Sampling design, observations of Euphydryas aurinia and management of the sites Kriegberg und Jonastal. Table S1. Weather parameters at the survey dates, measured at the DWD weather station Eisenach (DWD 2024). Table S2. Total number of marked individuals of Euphydryas aurinia, percentage of sexes and number of recaptured individuals per study site. [file ECE3-14-e70459-s001.docx]

**Appendix**

**Large, but dispersal-limited populations of the Marsh Fritillary *Euphydryas aurinia* persist on abandoned military training areas three decades after the end of the cold war**

Cindy Schröer, David Singer, Johannes Kamp

**Table A1**: Weather parameters at the survey dates, measured at the DWD weather station Eisenach (DWD 2024). Values show the mean/sum during the sampling periods (9 am to 5 pm).

| Survey date | Duration of sunshine (h) | Average air temperature at 2 m height (°C) | Average windspeed (km/h) |
| --- | --- | --- | --- |
| 16.05.2020 | 7.6 | 14.83 | 8.6 |
| 17.05.2020 | 8.1 | 15.2 | 9.5 |
| 21.05.2020 | 9.0 | 19.9 | 9.4 |
| 22.05.2020 | 0.4 | 21.6 | 11.4 |
| 26.05.2020 | 3.4 | 15.3 | 4.9 |
| 27.05.2020 | 6.7 | 19.7 | 7.2 |
| 30.05.2020 | 5.0 | 15.6 | 20.2 |
| 31.05.2020 | 6.0 | 15.9 | 13.6 |
| 02.06.2020 | 8.7 | 21.8 | 11.0 |
| 03.06.2020 | 3.8 | 21.3 | 9.7 |
| 08.06.2020 | 5.7 | 17.6 | 7.9 |
| 09.06.2020 | 0.0 | 16.8 | 10.2 |

**Table A2**: Total number of marked individuals of *E. aurinia,* percentage of sexes and number of recaptured individuals per study site.

| **Study site** | **Sex** | **Total** | **Percentage** | **1 recapture** | **2 recaptures** | **3 recaptures** |
| --- | --- | --- | --- | --- | --- | --- |
| Jonastal | Female | 16 | 28% | 0 | 0 | 0 |
|  | Male | 38 | 67% | 3 | 0 | 0 |
|  | Unknown | 3 | 5% | 0 | 0 | 0 |
|  | Total | 57 | 100% | 3 | 0 | 0 |
|  |  |  |  |  |  |  |
| Kriegberg | Female | 26 | 17% | 6 | 0 | 0 |
|  | Male | 112 | 73% | 11 | 1 | 1 |
|  | Unknown | 16 | 10% | 1 | 0 | 0 |
|  | Total | 154 | 100% | 18 | 1 | 1 |
|  |  |  |  |  |  |  |
| Hainich | Female | 670 | 30% | 38 | 2 | 0 |
|  | Male | 1489 | 67% | 124 | 6 | 0 |
|  | Unknown | 48 | 2% | 2 | 0 | 0 |
|  | Total | 2207 | 100% | 164 | 8 | 0 |


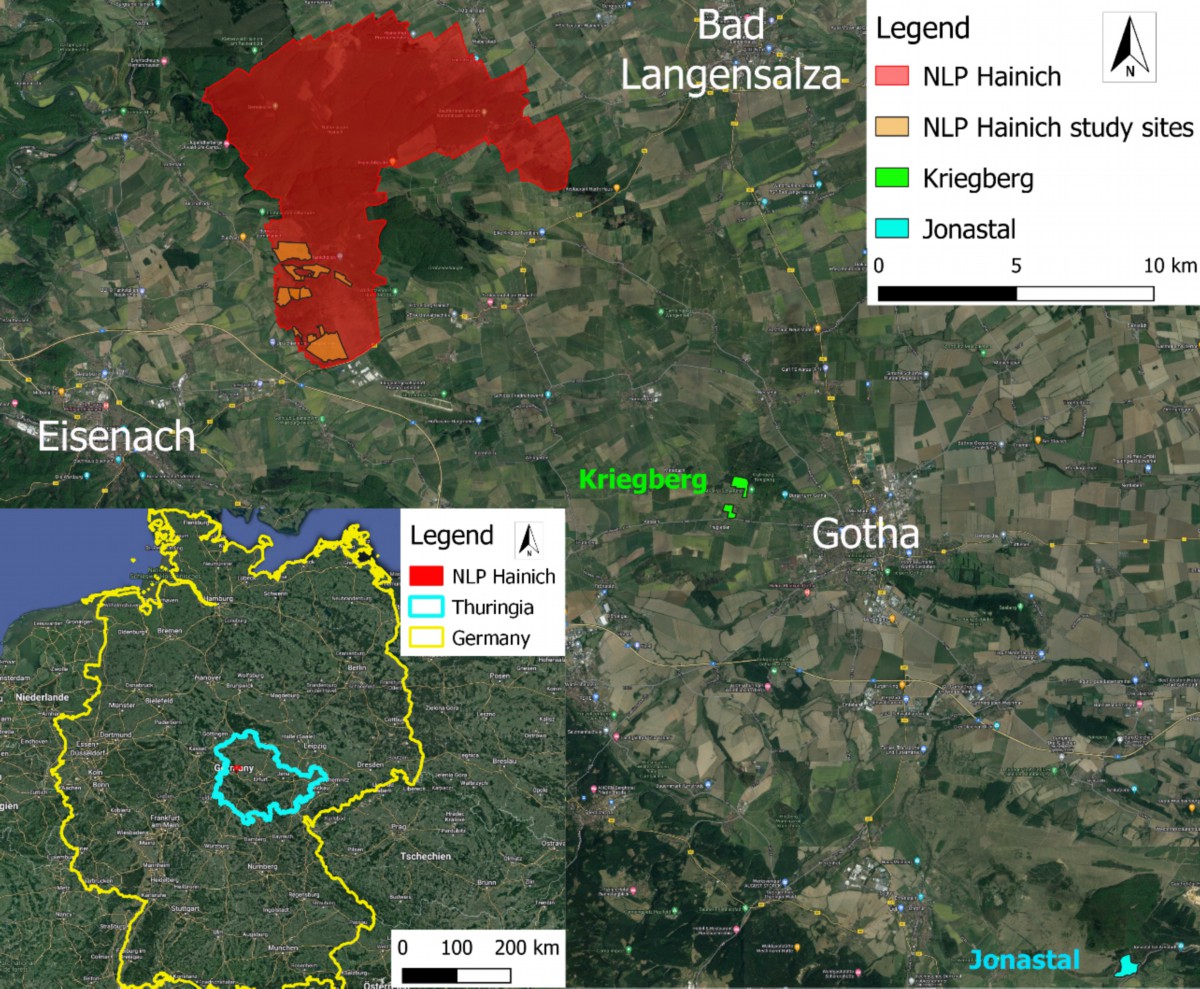


**Figure A1:** Overview of the location of the three study areas in Thuringia. Background map: (c) Google Maps.


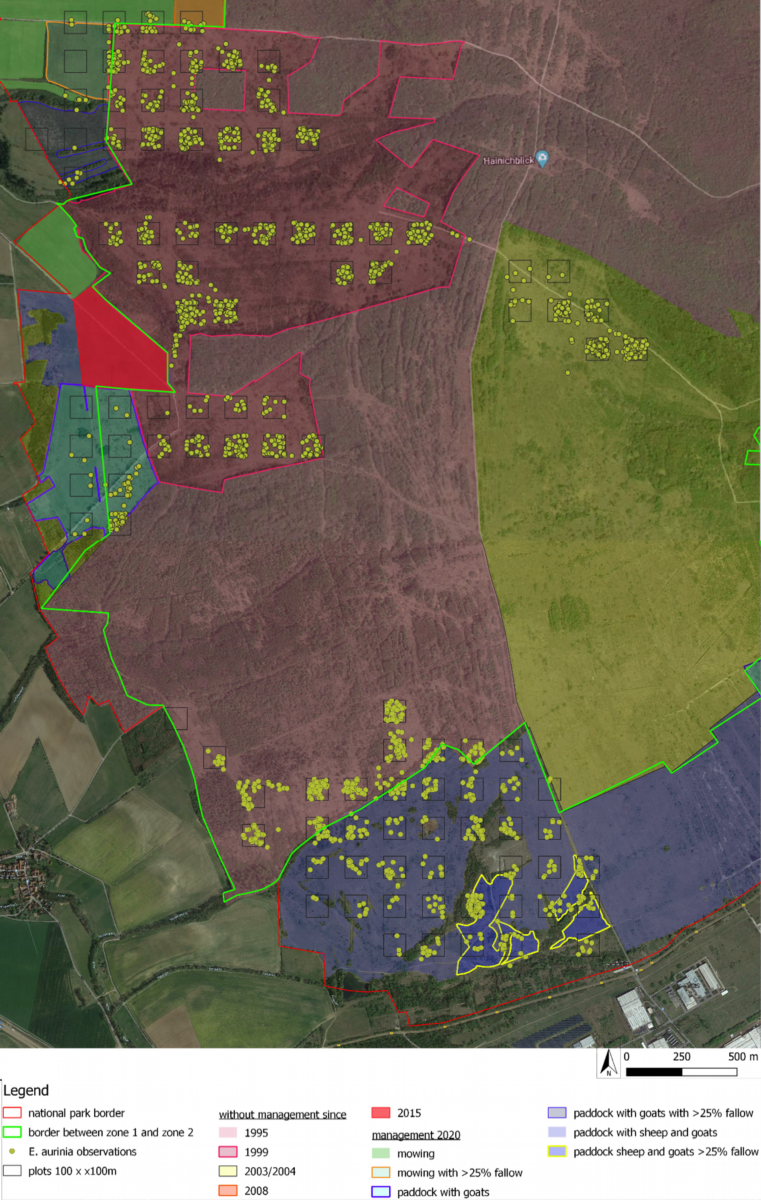


**Figure A2:** Sampling design, observations of E. aurinia and management of the National Park “Hainich”. Background map: (c) Google Maps.

**
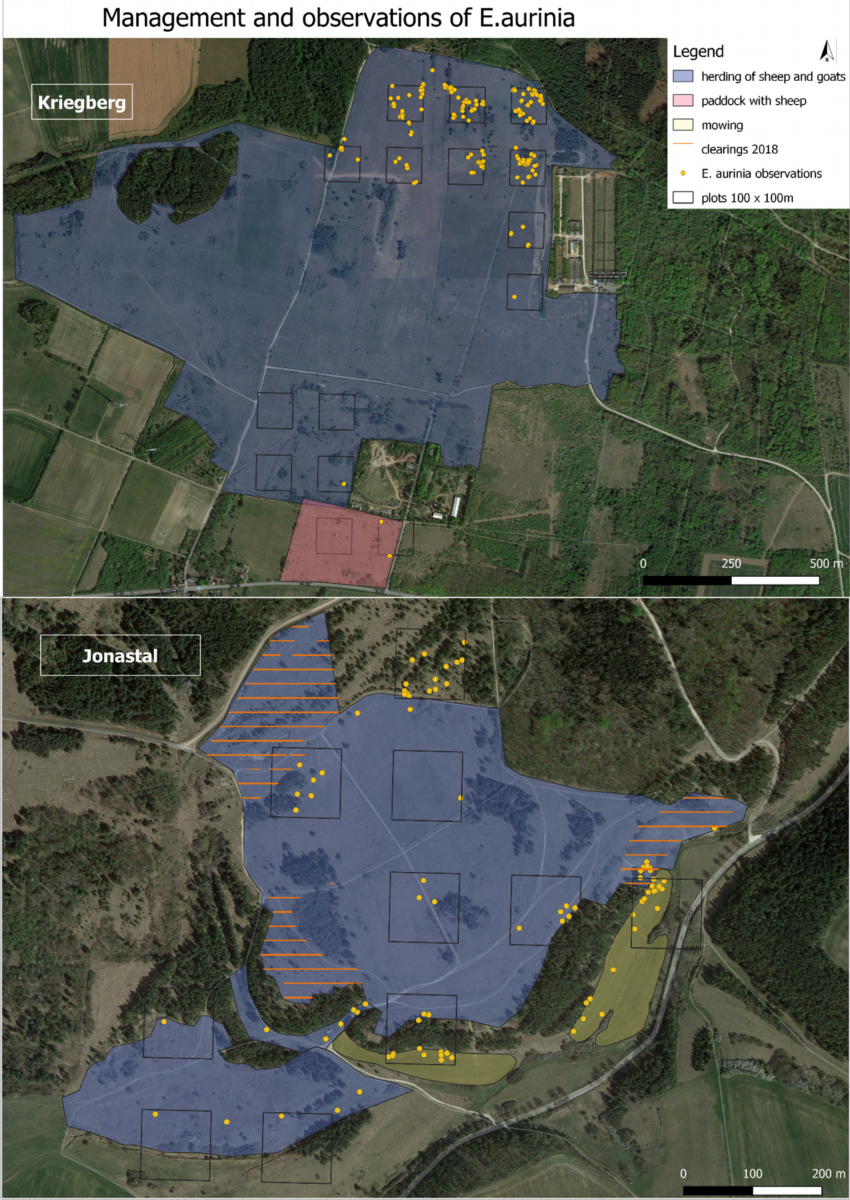
**

**Figure A3:** Sampling design, observations of E. aurinia and management of the sites Kriegberg und Jonastal. Background map: (c) Google Maps
